# CA9 transcriptional expression determines prognosis and tumour grade in tongue squamous cell carcinoma patients

**DOI:** 10.1111/jcmm.15252

**Published:** 2020-04-16

**Authors:** Chenyu Guan, Daiqiao Ouyang, Yongjie Qiao, Kan Li, Guangsen Zheng, Xiaomei Lao, Sien Zhang, Guiqing Liao, Yujie Liang

**Affiliations:** ^1^ Department of Oral and Maxillofacial Surgery Guanghua School of Stomatology Hospital of Stomatology Sun Yat‐Sen University Guangzhou China; ^2^ Guangdong Province Key Laboratory of Stomatology Guangzhou China

**Keywords:** bioinformatics, CA9, TCGA, tongue squamous cell carcinoma, tumour grade

## Abstract

CA9 is a member of the carbonic anhydrases’ family, that is often expressed in cancer cells under hypoxic condition. However, the role of CA9 in the molecular mechanisms of tongue squamous cell carcinoma (TSCC) pathogenesis remains unclear. CA9 expression was analysed using the TCGA database, and its influence on survival was performed using Kaplan‐Meier, LASSO and COX regression analyses. The correlation between CA9 and immune infiltration was investigated by CIBERSORT and ESTIMATE. Moreover, the relationship between CA9 expression and downstream molecular regulation pathways was analysed by GSEA, GO and WGCNA. CA9 expression correlated with clinical prognosis and tumour grade in TSCC. Moreover, CA9 expression potentially contributes to the regulation of cancer cell differentiation and mediates tumour‐associated genes and signalling pathways, including apoptosis, hypoxia, G2M checkpoint, PI3K/AKR/mTOR signalling and TGF‐beta signalling pathways. However, the follicular helper T cells, regulatory T cells, immune and stromal scores showed no significance between high and low CA9 expression groups. These findings suggested that CA9 plays a critical role of TSCC prognosis and tumour grade. CA9 expression significantly correlated with the regulation of cell differentiation, various oncogenes and cancer‐associated pathways.

## 1 INTRODUCTION

Tongue squamous cell carcinoma (TSCC) is one of the most common types of head and neck cancers, characterized by poor prognosis. Tumour recurrence and metastasis are the key reasons for the related worse survival rate.^1^ The analysis of biomarkers and associated molecular mechanisms provided a new way for the prediction of cancer patients’ prognosis. Various genes, such as the carbonic anhydrase 9 (CA9), have been found to relate to TSCC survival outcome. CA9 is a member of the carbonic anhydrases family, that is often expressed in cancer cells and under hypoxic conditions.^2^ CA9 has been found to play a critical part in the metastasis, recurrence and resistance of many cancers, including, clear cell renal cell carcinoma, hepatocellular carcinoma and prostate cancer.^3^, ^4^, ^5^ Recent studies indicated that the hypoxic condition of the cancer microenvironment regulates the expression CA9. Moreover, CA9 targeting, with immune‐checkpoint blockade, improves the response and survival of patients with hypoxic solid malignancies.^6^ In response to hypoxia, pancreatic cancer cells, that express activated KRAS, increase the expression of CA9 by stabilizing the transcription factor HIF1A and HIF2A, and mediate pH and glycolysis in the tumour microenvironment.^7^ Eckert et al^8^ demonstrated that CA9 levels in oral squamous cell carcinoma (OSCC) samples were higher than those in precancerous tissues. Additionally, immunohistochemical analysis of CA9 expression, in oral lichen and leukoplakia, suggested that its expression was also observed in oral precancerous lesions.^9^, ^10^ CA9 plasma levels may be used as a non‐invasive method for monitoring OSCC progression.^11^ LCN2 was able to target the CA9 transcriptional and post‐transcriptional regulation in OSCC cells, which suppressed metastasis.^12^

However, the molecular mechanism and regulation of CA9 gene expression in TSCC still require further investigation. The main goal of our study was focused on investigating the mechanism by which CA9 expression influences TSCC progression. Our results indicated that CA9 high expression was related to TSCC patients’ poor outcome and that was mediated by oncogenes and tumour‐associated pathways. In addition to the role of CA9 in hypoxia, we found that CA9 had a significant correlation with TSCC tumour grade and cell differentiation.

## 2 MATERIALS AND METHODS

### 2.1 Data source

Expression data and corresponding clinical information of TSCC patients were downloaded from The Cancer Genome Atlas (TCGA) official website, including other and unspecified parts of tongue and base of tongue (tumour samples: n = 146, normal samples: n = 15, fragments per kilobase million [FPKM]).

### 2.2 Transcriptional expression of carbonic anhydrases

An expression matrix of the carbonic anhydrases CA1, CA2, CA3, CA3‐AS1, CA4, CA5A, CA5AP1, CA5B, CA5BP1, CA6, CA7, CA8, CA9, CA10, CA11, CA12, CA13, CA14, CA15P1 and CA15P2 was formed with FPKM of each mRNA expression in the TSCC samples. The heatmaps were generated with the ‘pheatmap’ package in R (R version 3.5.3).

### 2.3 Prognostic analysis of carbonic anhydrases

The prognostic analysis of TSCC patients was evaluated using the Kaplan‐Meier method, the univariate Cox proportional hazards regression and the multivariate Cox regression analysis. The samples were stratified as high or low expression around the median of each carbonic anhydrases’ expression (high expression: n = 73, low expression: n = 73).

### 2.4 Identification of CA9 differentially expressed genes (DEGs)

DEGs were identified in TSCC tissues by comparison with CA9 high and low expression groups and using the ‘edgeR’ package in R (R version 3.5.3). The heatmap and volcano plot, with clustering for the significantly differentially expressed mRNAs in TSCC between CA9 high and low expression groups, were generated with the ‘ggplot2’ package. GO analysis was constructed by DAVID 6.8.^13^, ^14^

### 2.5 Gene set enrichment analysis (GSEA)

The signalling pathway, underlying the association between CA9 expression levels, was explored with GSEA (version 3.0.). The number of permutations was set at 1000, and the *P‐*value < .05 and an FDR < 0.25 were considered statistically significant. Multiple GSEA plots were produced by ‘plyr’, ‘ggplot2’ and ‘grid’ packages.

### 2.6 The clinical features correlated with of CA9 expression level

The samples with insufficient clinical data were filtered out. Filtered clinical information from TSCC patients, including age, gender, tumour grade and stage, was obtained from TCGA. Patients were divided into two groups and according to the median of CA9 expression level (high expression: n = 63, low expression: n = 62). COX regression and nomogram were used to analyse the role of CA9 in TSCC patients’ clinical characteristics. Based on the C‐index values, a nomogram integrating group (high expression and low expression of CA9), gender, grade, stage were constructed. The total points were calculated by adding the points of the CA9 expression level, gender, grade and stage. The calibration curve for predicting 3‐ and 5‐year OS indicated that the nomogram‐predicted survival closely corresponded with actual survival outcomes.

### 2.7 Immune landscape related to CA9 expression level

CIBERSORT is a method for characterizing the immune cell composition using gene expression data to define 22 immune cell subtypes.^15^ The results include a *P‐*value for each sample of global deconvolution. Each sample in the data set will get a *P*‐value, that is used to select the samples for further studies if their *P‐*values are less than .05. The immune and stromal scores were calculated by ESTIMATE.^16^, ^17^

### 2.8 Weighted gene co‐expression network analysis

The WGCNA^18^ package was used to identify key modules, associated with tumour grade and based on CA9 expression levels. The module eigengenes of the clinical features were hierarchically clustered into different colour modules. GO analysis was used to evaluate the molecular functions of the co‐expressed genes. Hub genes, that are related to tumour grade, were identified by Cytoscape.

### 2.9 Statistical analysis

The analyses were carried out using the ‘R’ software (version 3.5.3), GraphPad Prism 8 and IBM SPSS Statistics 19. *P* < .05 was considered statistically significant. ***P < .001, **P < .01 *P < .05.

## 3 RESULTS

### 3.1 Evaluating the prognostic significance of carbonic anhydrases in TSCC

**Figure 1:**
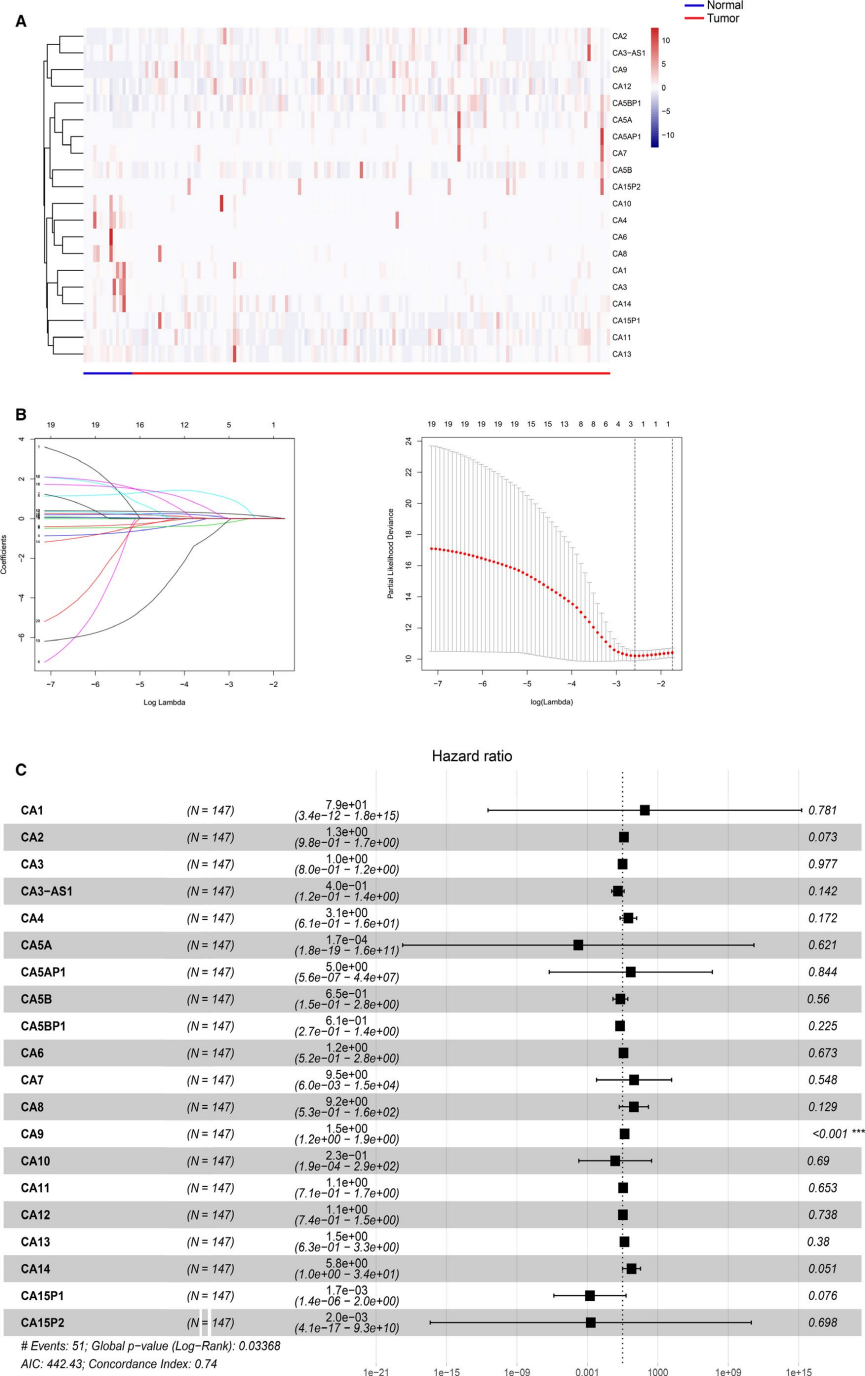
Evaluating the prognostic significance of carbonic anhydrases in TSCC. A, Heatmap of carbonic anhydrases expression in TSCC. B, The validation and elucidation of carbonic anhydrases was performed through the LASSO regression model. C, The forest plot exhibited hazard ratios (HR) and 95% confidence interval (95% CI) by multivariate Cox regression analysis

The expression levels of carbonic anhydrases are shown in Figure 1A. Twenty anhydrases, that are related to prognosis and overall survival prediction, were identified using LASSO Cox analysis and univariate and multivariate Cox regression analyses (Table 1; Figure 1B,C). CA9 was eventually identified based on the Kaplan‐Meier survival analysis (*P* = .002; Table 1).

**Table 1:** Univariate analysis and Kaplan‐Meier survival analysis of carbonic anhydrases in TSCC

Gene	Univariate analysis		Kaplan‐Meier survival analysis
	HR	*P*‐value	*P*‐value
CA9	1.386	<.001^***^	.002^**^
CA5B	0.388	.106	.009^**^
CA5AP1	0.478	.835	.056
CA4	4.279	.030^*^	.061
CA12	1.198	.164	.181
CA5A	0.059	.781	.371
CA2	1.077	.373	.384
CA5BP1	0.567	.120	.400
CA8	0.894	.892	.432
CA7	0.968	.982	.565
CA6	1.765	.186	.567
CA10	0.046	.467	.583
CA1	0.013	.694	.617
CA14	1.557	.553	.631
CA3‐AS1	0.793	.511	.632
CA3	1.011	.906	.725
CA11	0.901	.585	.757
CA13	0.967	.909	.775
CA15P1	0.108	.366	.785
CA15P2	0.009	.677	.864

*P* < .05 was considered statistically significant.

^*^ *P* < .05.

^**^ *P* < .01.

^***^ *P* < .001.

### 3.2 Analysis of the differentially expressed genes and immune infiltration level with CA9 high and low expression group in TSCC

**Figure 2:**
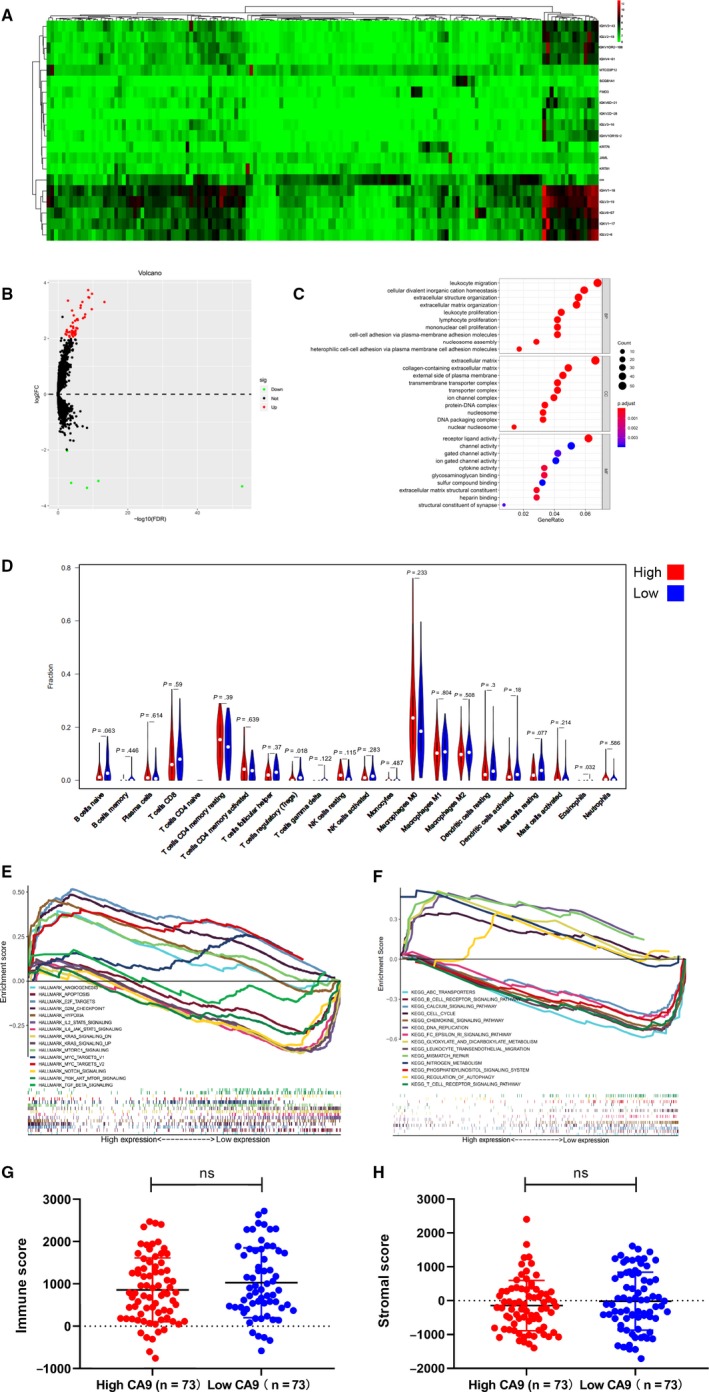
Analysis of differentially expressed genes and immune infiltration level with CA9 high and low expression groups in TSCC. A, Heatmap of the analysed differentially expressed genes. B, Volcano plot of the differentially expressed genes with CA9 high and low expression groups. C, Go analysis of the differentially expressed genes with CA9 high and low expression groups. D, Analysis of Hallmark gene sets by GSEA. E, Analysis of KEGG by GSEA. F, Violin plot of immune infiltration level between CA9 high and low expression groups. G, Immune scores between CA9 high and low expression groups. H, Stromal scores between CA9 high and low expression groups

A total of 940 differentially expressed genes were screened with CA9 high and low expression groups. The top twenty differentially expressed genes are shown in the heatmap (Figure 2A). The GO analysis suggested that the differentially expressed genes were closely associated with leucocyte migration, extracellular matrix and receptor ligand activity (Figure 2C, Supplementary Table 1). In the Hallmarks gene sets, the highly enriched I and significant pathways were E2F targets, apoptosis, hypoxia, G2M checkpoint, PI3K/AKR/mTOR signalling pathway and TGF‐beta signalling. In the KEGG gene sets, pathways, such as nitrogen metabolism, mismatch repair, glyoxylate and dicarboxylate metabolism, DNA replication and ABC transporters, were identified (Figure 2D,E).

The CIBERSORT analysis demonstrated that follicular helper T cells, regulatory T cells and eosinophils were significantly infiltrated in the different groups (Figure 2F). However, the immune score and stromal score showed no significance in the high and low CA9 expression groups (Figure 2G,H).

### 3.3 Clinical characteristics and risk scores of CA9 in TSCC

**Figure 3:**
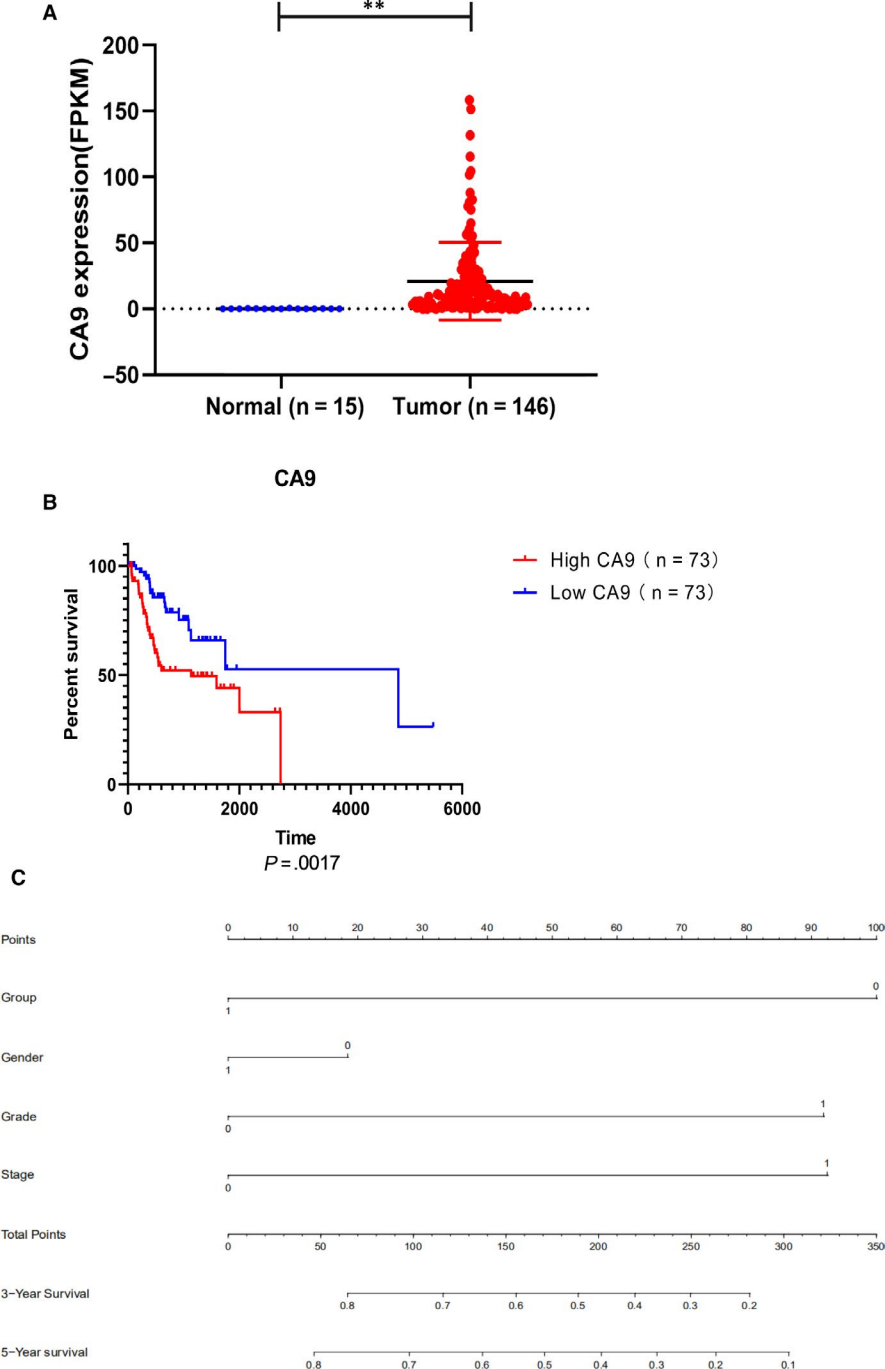
CA9 clinical characteristics and risk scores in TSCC. A, Scatter plots showing the expression levels of CA9 between normal tissue (blue) and TSCC tumour tissue (red). B, Kaplan‐Meier survival analysis of the role of CA9 in the overall survival of head and neck cancer patients. C, Nomogram for predicting 3‐y or 5‐y survival, in relation to CA 9 expression in TSCC patients

CA9 expression levels are shown in Figure 3A. The transcription levels of CA9 in the tumours were significantly higher than those in normal tissues. According to the Kaplan‐Meier analysis method, a high expression level of CA9 in TSCC was associated with poorer overall survival (*P* = .0017; Figure 3B). Samples, containing complete clinical information (age, gender, grade and stage), were divided into groups of low and high around the median of each CA9 expression. Univariate and multivariate Cox regression analyses indicated that CA9 expression correlates with tumour grade (Table 2). In view of the prognostic value of CA9 in TSCC, a nomogram was constructed for predicting 3‐ and 5‐year survival. The result illustrated that CA9 expression (group) shares the largest contribution to overall survival, followed by the grade and stage (Figure 3C).

**Table 2:** Univariate and multivariate analyses of the clinical characteristics related to the expression level of CA9 in TSCC

Parameters	Univariate analysis		Multivariate analysis			
	HR	*P*‐value	HR	95% CI		*P*‐value
Group (high vs low)	0.410	.006^**^	0.403	0.212	0.766	.006^**^
Age (≤60 vs >60)	1.153	.640	1.072	0.584	1.967	.823
Gender (female vs male)	0.802	.492	0.854	0.450	1.618	.628
Grade (I + II vs III + IV)	2.041	.032^*^	2.293	1.187	4.431	.014^*^
Stage (I + II vs III + IV)	2.161	0.062	2.319	1.028	5.229	0.043^*^

*P* < .05 was considered statistically significant.

^*^ *P* < .05.

^**^ *P* < .01.

### 3.4 Identify key modules associated with patients’ grade based on the expression levels of CA9 in TSCC

**Figure 4:**
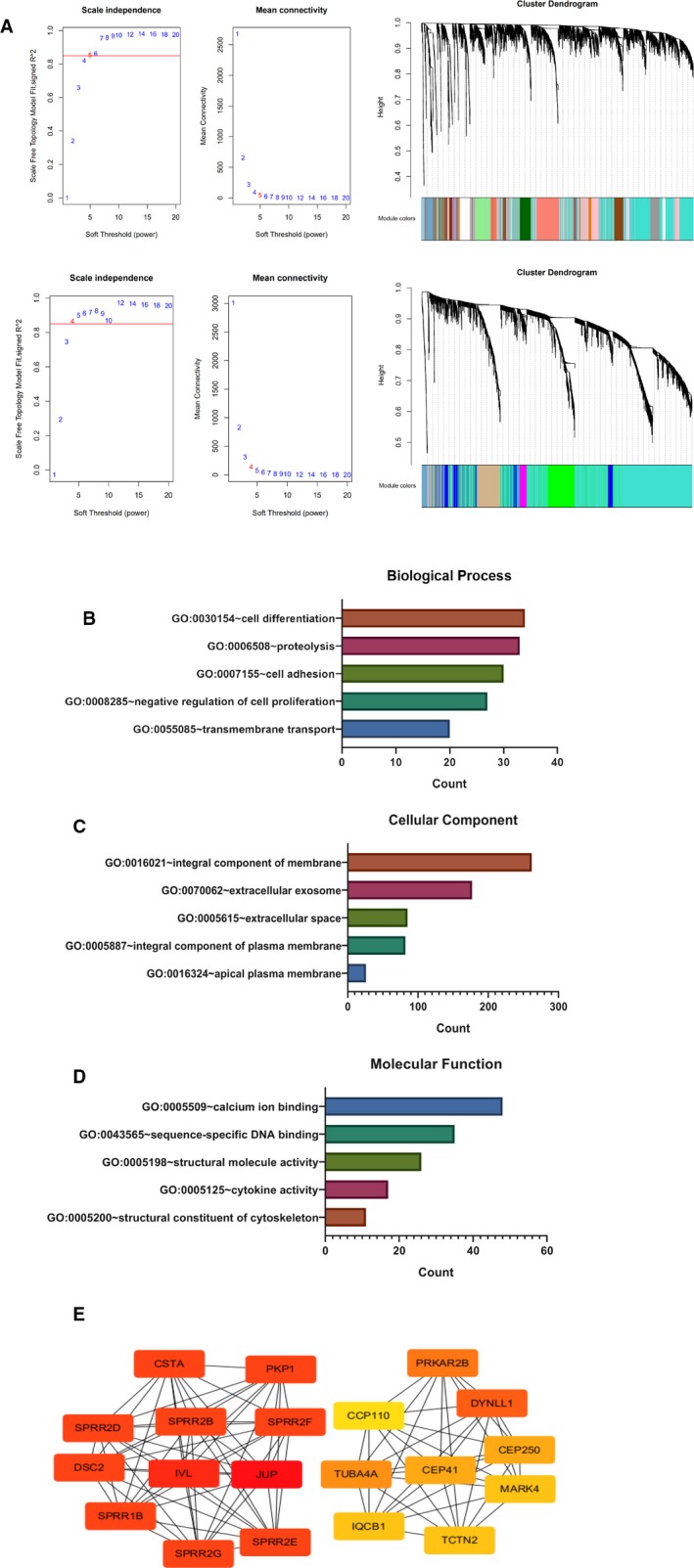
Grade weighted gene co‐expression network analysis of module eigengenes of grade correlated with CA9 expression in TSCC. A, The clustering dendrograms and the scale‐free topology model of the CA9 high and low expression groups. B, Biological process enrichment of module eigengenes of grade correlated with CA9 expression. C, Cellular component enrichment of module eigengenes of grade correlated with CA9 expression. D, Molecular function of grade module eigengenes correlated with CA9 expression. E, Hub genes protein interaction network of the significant modules for patients’ grade

Based on the results shown in Table 2, the weighted gene co‐expression network analysis was used to identify the module eigengenes and clinical traits that are related to tumour grade. Gene modules were analysed in the CA9 high and low expression groups, respectively. In the high expression group, soft power 5 and a minimum module size cut‐off of 30 were chosen as the threshold to identify co‐expressed gene modules. The soft power of the low expression group was 4 (Figure 4A). The module‐feature relationship was found between tumour grade and 19 different modules and therefore was chosen for subsequent analyses. A total of 929 genes, with biological relevance, were investigated using DAVID database for GO (Gene Ontology) analyses (Figure S1). The genes were closely correlated with the integral component of the membrane, through the analysis of the cellular component. The molecular function enrichment showed that these genes were mainly related to calcium ion binding (Figure 4B‐D). Hub genes that were associated with grade, such as JUP, IVL, DSC2, SPRR1B, SPRR2D, SPRR2B, SPRR2F, SPRR2G, SPRR2E, CSTA, PKP1 and DYNLL1, were identified (Figure 4E).

## 4 DISCUSSION

Carbonic anhydrase 9 is a type of zinc metalloenzyme that is able to catalyse the reversible hydration of carbon dioxide.^19^ It can control the intracellular pH and protect cancer cells from hypoxia‐induced apoptosis.^20^ In many malignancies, CA9 is highly related to hypoxia and is regulated by the transcription factor HIF‐1α.^21^ Knockdown of CA9 abolished the chemoresistance resulting from ZEB1 overexpression and prevented maintenance of pHi mediated by overexpression of ZEB1 in tongue cancer cells. ZEB1 and CA9 protein expression was associated with a poor prognosis for patients in TSCC (Table S2).^22^ Meanwhile, high CA9 levels reduce the chemosensitivity and facilitate the survival of various cancer cells by enhancing the acidification of the cancer microenvironment.^23^ In renal clear cell carcinoma, CA9 could be an independent prognostic tumour marker and a diagnostic biomarker. Similarly, our analysis suggested that CA9 expression level was associated with prognosis and could regulate signalling pathways in TSCC, including hypoxia and cell cycle. In oral squamous cell carcinoma, a high CA9 mRNA level was associated with an increased risk of locoregional recurrence, while the expression level of CA9 was related to tumour grade and clinical stages in TSCC patients.^24^ However, TCGA data showed that there was no significant difference between the mRNA expression level of CA9 and the prognosis of patients with cancers occurring in the lips and the other sites of oral cavity (Table S2). Therefore, our research suggested that CA9 could be used as a potential predictor of tumour grade and prognosis in TSCC.

The prognostic landscape of the carbonic anhydrase was first analysed in TSCC. Interestingly, CA9 expression levels were significantly associated with the survival outcome of TSCC patients using the TCGA cohort and analysed by univariate Cox regression and Kaplan‐Meier analysis (Table 1). Multivariate Cox proportional hazards regression also demonstrated a significant correlation between CA9 expression level and prognosis in TSCC patients. Recent evidence confirmed that CA9 was able to mediate the survival of oral squamous cell carcinoma patients and could be an independent prognostic factor.^8^ The analysis of the clinical features of the TSCC TCGA cohort showed that CA9 expression levels correlate with patients’ tumour grade. These results suggest that CA9 may be used as a potential diagnostic and prognostic factor in TSCC.

The comparison between CA9 high and low expression groups showed that the differentially expressed genes were enriched in leucocyte migration and proliferation. CA9 also plays a role in lymphocytes and mononuclear cell proliferation. Additionally, the GSEA analysis showed that the CA9 expression level was negatively related to the leucocyte transendothelial migration and T cell receptor signalling pathway. In the CIBERSORT result, CA9 high expression level significantly inhibited the infiltration level of follicular helper and regulatory T cells, which is probably due to the decreased proliferation and migration of T cells. Previous studies have reported that CA9 encodes HLA class I‐ and HLA class II‐restricted epitopes that could be recognized by helper T cells in renal cell carcinoma.^25^ The regulation of T cells might also be due to the expression of CA9‐specific protein epitopes in TSCC.

Using COX regression model, we found that CA9 was highly related to tumour grade in tongue cancer patients. Thus, we analysed tumour grade co‐expressed genes in high and low expression groups using WGCNA. We found that genes, which correlated with tumour grade, were highly enriched in cell differentiation and negative regulation of cell proliferation using GO analysis. In sinonasal papilloma, CA9 expression correlated with cancer cells differentiation and proliferation and emerged as a marker of cancer recurrence.^26^ In pancreatic ductal adenocarcinoma, CA9 expression was significantly lower in cases with well‐differentiated adenocarcinoma.^27^ It is possible that CA9 influenced TSCC grade by regulating neoplastic cell differentiation and proliferation. In addition, hug genes, such as DSC2, CSTA, PKP1 and DYNLL1, also appear to be involved in this process. In the esophageal squamous cell carcinoma, DSC2 displayed a role in tumour differentiation, metastasis and patients’ clinical outcomes.^28^ PKP1 was inversely associated with the histological grade of human primary oropharyngeal squamous cell carcinoma.^29^ It could be hypothesized that the role of CA9 in cancer cell differentiation may also be a result of the activation of multiple co‐expressed genes that affect TSCC differentiation.

In conclusion, CA9 high expression correlates with poor prognosis and tumour grade in TSCC. The activation of tumour‐associated pathways and cell differentiation‐related genes plays a crucial part in these biological processes. To test these hypotheses, it will be necessary to undertake cytological and animal experiments studies in the future. These investigations are currently under way.

## CONFLICT OF INTEREST

We have no conflicts of interest.

## AUTHOR CONTRIBUTIONS

Guan Chenyu and Daiqiao Ouyang involved in most of the experiments, collected and analysed data, and drafted the manuscript. ^*^Yongjie Qiao, Kan Li, Guangsen Zheng, Xiaomei Lao and Sien Zhang assisted in analysed data and drafted the manuscript. Guiqing Liao and Yujie Liang conceived and designed the experiments, oversaw the collection of results, data interpretation and wrote the manuscript. All authors read and approved the paper.

## Supporting information


**Figure S1**

**Table S1**

**Table S2**

## Data Availability

All data included in this study are available upon request by contact with the corresponding author.
