# Supplementary figures and images for: CA9 transcriptional expression determines prognosis and tumour grade in tongue squamous cell carcinoma patients
Source: J Cell Mol Med. 2020 Apr 16;24(10):5832–41. doi: 10.1111/jcmm.15252 (PMC7214172; doi:10.1111/jcmm.15252)

# Module-trait relationships

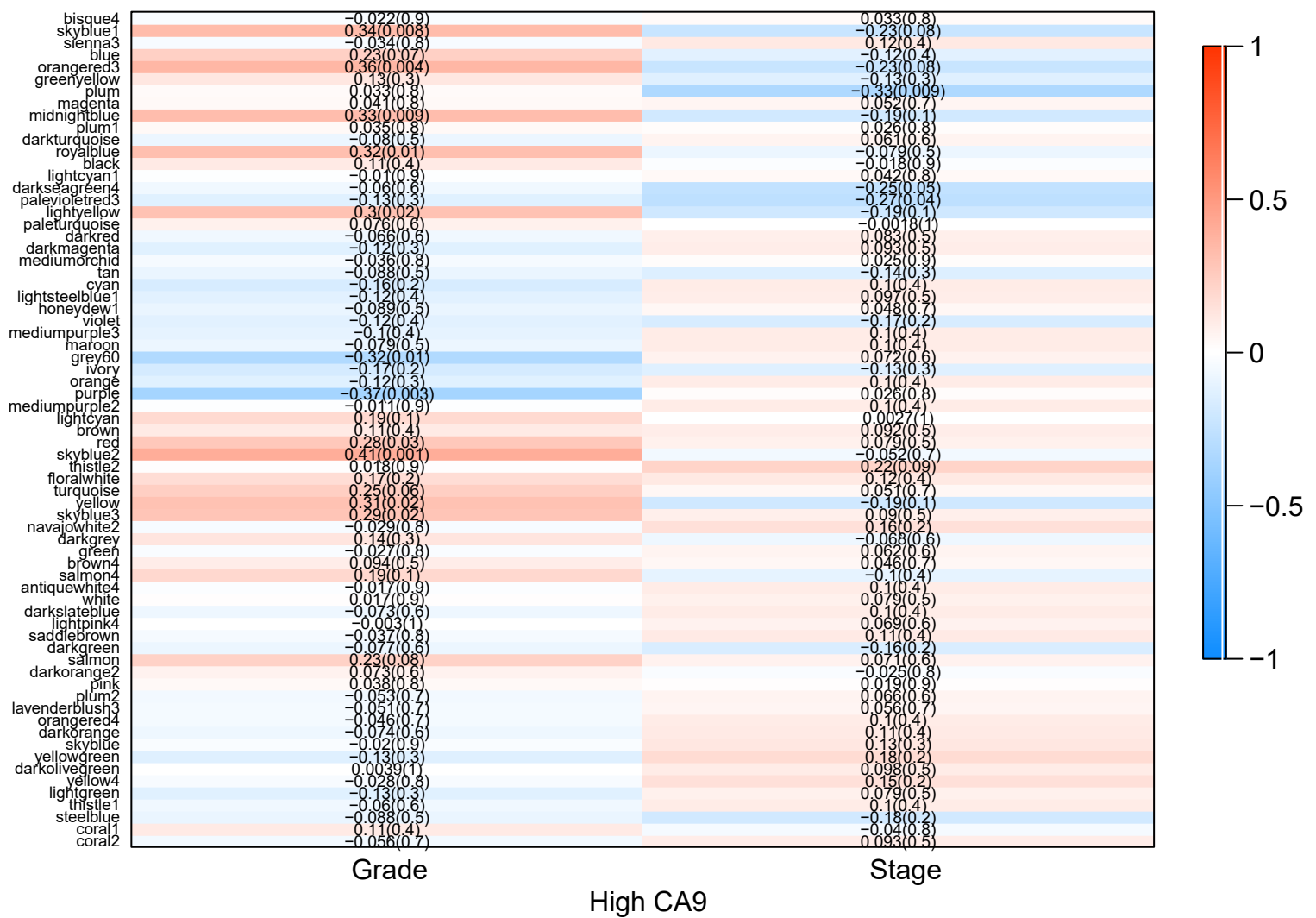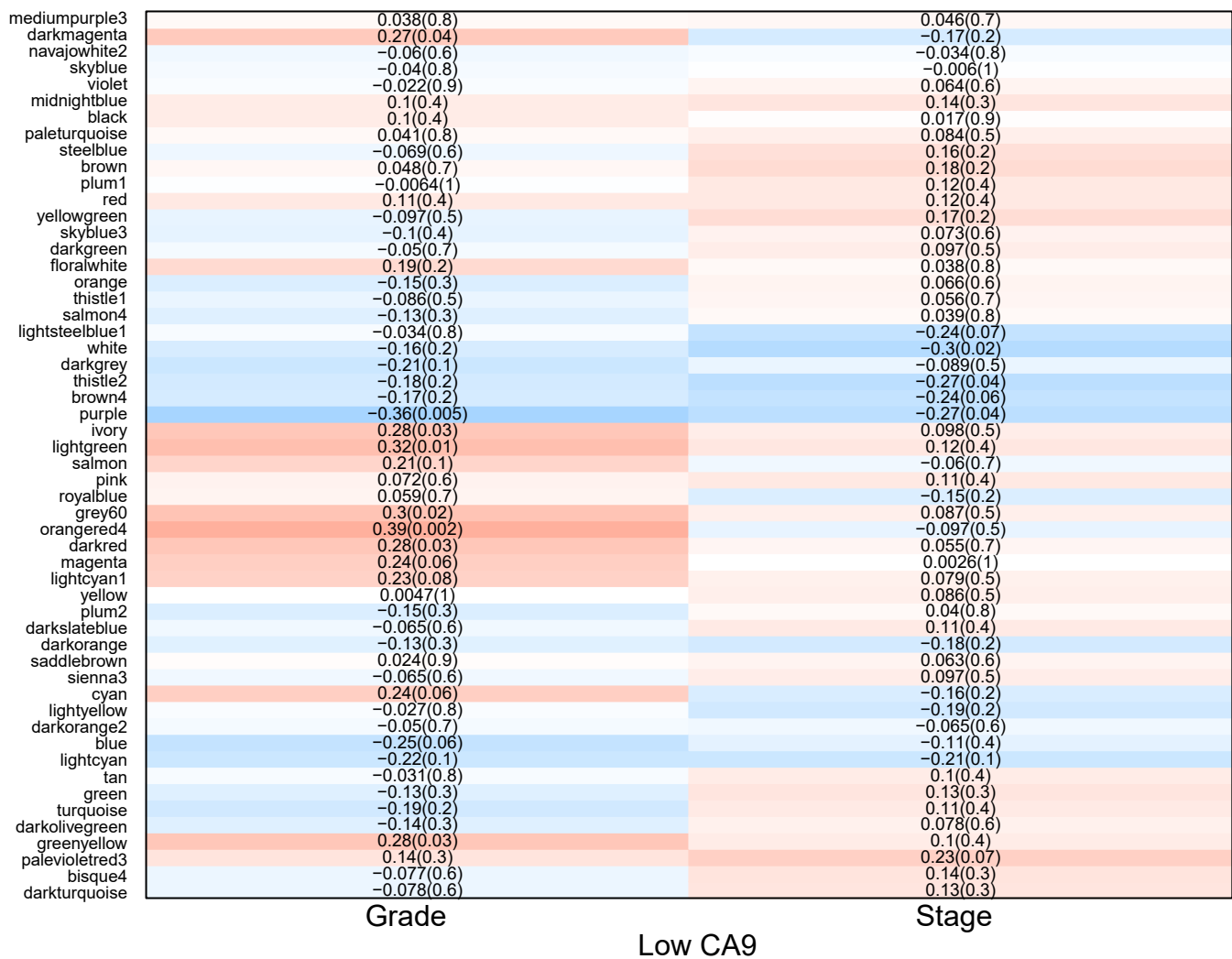

Supplement: Supplementary file 1 — Figure S1 [file JCMM-24-5832-s001.pdf]
