# Supplementary material for: CA9 transcriptional expression determines prognosis and tumour grade in tongue squamous cell carcinoma patients
Source: J Cell Mol Med. 2020 Apr 16;24(10):5832–41. doi: 10.1111/jcmm.15252 (PMC7214172; doi:10.1111/jcmm.15252)
Supplement: Supplementary file 3 — Table S2 [file JCMM-24-5832-s003.docx]

**Supplemental Table 2**

Univariate analysis and Kaplan-Meier survival analysis of carbonic

| **[Tumor](C:/Users/chenyu/AppData/Local/youdao/dict/Application/8.5.3.0/resultui/html/index.html#/javascript:;) [site](C:/Users/chenyu/AppData/Local/youdao/dict/Application/8.5.3.0/resultui/html/index.html#/javascript:;)** | **Univariate analysis** | | **Kaplan-Meier**  **survival analysis** |
| --- | --- | --- | --- |
|  | HR | *p*-value | *p*-value |
| Tongue | 1.386 | ＜0.001*** | 0.002** |
| Lip and the other parts of oral cavity | 0.94 | 0.46 | 0.7704 |

anhydrases in different [tumor](C:/Users/chenyu/AppData/Local/youdao/dict/Application/8.5.3.0/resultui/html/index.html#/javascript:;) [site](C:/Users/chenyu/AppData/Local/youdao/dict/Application/8.5.3.0/resultui/html/index.html#/javascript:;) of oral cavity
